# The light-from-above prior is intact in autistic children

**DOI:** 10.1016/j.jecp.2017.04.005

**Published:** 2017-09

**Authors:** Abigail Croydon, Themelis Karaminis, Louise Neil, David Burr, Elizabeth Pellicano

**Affiliations:** a Centre for Research in Autism and Education (CRAE), Department of Psychology and Human Development, UCL Institute of Education, University College London, London WC1H 0NU, UK; b Centre for Language Studies, Radboud University, Erasmusplein 1, 6525 HT Nijmegen, The Netherlands; c Institute of Neuroscience, National Research Council (CNR), 56100 Pisa, Italy; d School of Psychology, University of Western Australia, Crawley, Perth, Western Australia 6009, Australia

**Keywords:** Perception, Autism, Bayesian priors, Light-from-above, Development, Bias

## Abstract

Sensory information is inherently ambiguous. The brain disambiguates this information by anticipating or predicting the sensory environment based on prior knowledge. Pellicano and Burr (2012) proposed that this process may be atypical in autism and that internal assumptions, or “priors,” may be underweighted or less used than in typical individuals. A robust internal assumption used by adults is the “light-from-above” prior, a bias to interpret ambiguous shading patterns as if formed by a light source located above (and slightly to the left) of the scene. We investigated whether autistic children (*n* = 18) use this prior to the same degree as typical children of similar age and intellectual ability (*n* = 18). Children were asked to judge the shape (concave or convex) of a shaded hexagon stimulus presented in 24 rotations. We estimated the relation between the proportion of convex judgments and stimulus orientation for each child and calculated the light source location most consistent with those judgments. Children behaved similarly to adults in this task, preferring to assume that the light source was from above left, when other interpretations were compatible with the shading evidence. Autistic and typical children used prior assumptions to the same extent to make sense of shading patterns. Future research should examine whether this prior is as adaptable (i.e., modifiable with training) in autistic children as it is in typical adults.

## Highlights

• Children interpreted shading patterns assuming a light source from above-left.
• Autistic and typical children used prior assumptions to a similar extent as adults.
• There were no increases in the level of bias between 7 years and adulthood.

## Introduction

In human vision, the complex dimensions of a visual scene—the shapes of objects, their spatial arrangement, and their material properties—are reduced to flat patterns of excitation of the cones and rods of the retina. Information entering the brain is inherently ambiguous, compatible with a range of interpretations. Visual input, therefore, is “underspecified” for the task of providing the reliable and stable awareness of the environment that we experience. Consequently, perception has long been considered as a process of “unconscious inference” (Helmholtz, 1866/1911), in which existing knowledge is spontaneously and automatically deployed to interpret the meaning of sensory signals. Expectations based on experience of how the material environment works—for example, of faces being convex or of light sources being overhead—feed into the construction of a percept by the brain.

The framework provided by Helmholz (1866/1911) was extended to characterize perceptual inferences as “hypotheses” or informed speculations using noisy and limited data (Gregory, 1980). Perceptual decisions are made possible by comparing the probability of the sensory evidence and prior experience. The Bayesian framework has since supplied an established mathematical model for perceptual decision making under conditions of uncertainty. In Bayesian terms, if both sensory signals and knowledge-based hypotheses are represented as probability distributions, techniques of statistical inference can be used to locate the combined point of maximal probability, the “best guess” interpretation (Knill, Kersten, & Yuille, 1996). In Bayesian perceptual inference, the prior probability distribution, or “prior,” represents a “baseline” understanding of the likelihood of particular environmental conditions on the basis of past experience (Gregory, 1980).

A number of visual priors have been established and are thought to improve overall perceptual efficiency by weighting perceptual hypotheses in a broadly reliable way. For example, a prior for convexity reflects the predominance of convex, over concave, objects in the world (Langer and Bülthoff, 2001, Sun and Perona, 1996). The statistical inference calculation implies a trade-off between the image data and the prior probability, such that perception will be more prior driven when ambiguity in the sensory input is high. In some circumstances, prior-driven expectations will be misguided, resulting in visual illusions. For example, perceiving a hollow mask as a convex mask implies the operation of overriding expectations of convexity in faces. Hence, when problems of object perception are resolved on a probabilistic basis, the optimal solution may still be “inaccurate” (Kersten & Yuille, 2003). Importantly, in the context of this research, Bayesian priors envisage dynamic connections among perceptual inference, experience of the environment, and behavior (Lee, Yang, Romero, & Mumford, 2002).

Atypicalities in sensation and perception are highly characteristic of autism (Baranek et al., 2006, Leekam et al., 2007, Mottron et al., 2006, Simmons et al., 2009). Although difficulties in social communication are considered hallmarks of autism, sensory reactivity, including hypersensitivity (e.g., to light or touch) and hyposensitivity (e.g., to pain), was noted during the first description of autism (Kanner, 1943). Sensory reactivity has since been shown to be present in the majority of autistic children and adults (Ben-Sasson et al., 2009, Leekam et al., 2007, Simmons et al., 2009), to be pervasive and persistent across development (Crane et al., 2009, McCormick et al., 2016), and to have a substantial impact on the lives of autistic people (e.g., Dickie et al., 2009, Grandin, 2009, Williams, 1994).

In the perceptual domain, the majority of scientific studies have reported atypical processing in aspects of visual perception and visual attention ranging from characteristically nonsocial stimuli and tasks, such as discrimination of chromatic stimuli (e.g., Franklin et al., 2010), cast shadow (Becchio, Mari, & Castiello, 2010), static gratings (e.g., Bertone, Mottron, Jelenic, & Faubert, 2005), moving dots (e.g., Milne et al., 2002, Pellicano et al., 2005), and complex objects (e.g., “Greebles”) (e.g., Behrmann et al., 2006, Davies et al., 1994), to social stimuli, including faces (e.g., Dalton et al., 2005, Kemner and van Engeland, 2006), eye gaze (e.g., Elsabbagh et al., 2009), and biological motion (e.g., Blake et al., 2003, Klin et al., 2009; for a review, see Simmons et al., 2009). Theories of autistic perception have explained these findings in terms of a “detail-focused” perceptual style (Frith & Happé, 1994), generally enhanced perceptual functioning (Mottron et al., 2006), or reduced generalization (Plaisted, 2001).

Building on these accounts, Pellicano and Burr (2012) suggested that it is not sensory processing itself that is atypical in autism but rather the *interpretation* of the sensory input. Specifically, drawing on the tools of Bayesian theory, they proposed that the internal priors of autistic people are underweighted or less used than in typical individuals. Attenuated priors might result in enhanced perception in some contexts and reduced performance in others, depending on whether the task draws more heavily on sensory input itself or on successful perceptual prediction using prior knowledge. This idea was expanded by several related Bayesian and neurobiological accounts that proposed possible atypicalities in predictive processing in autism (Brock, 2012, Friston et al., 2013, Lawson et al., 2014, Sinha et al., 2014, van Boxtel and Lu, 2013, Van de Cruys et al., 2014).

Evidence supporting the hypothesis of attenuated priors in autism comes from studies showing that autistic children and adults show reduced adaptation, a form of experience-dependent plasticity in which neural systems fine-tune to the current visual environment according to the previous context. There is now considerable evidence for reduced adaptation in autism for high-level visual attributes, both social stimuli (e.g., faces: Pellicano, Jeffery, Burr, & Rhodes, 2007; biological motion: van Boxtel, Dapretto, & Lu, 2016) and nonsocial stimuli (e.g., numerosity: Turi et al., 2015), as well as for other sensory modalities (e.g., touch: Tommerdahl, Tannan, Holden, & Baranek, 2008; audition: Lawson, Aylward, White, & Rees, 2015); audiovisual calibration: Turi, Karaminis, Pellicano, & Burr, 2016).

More recently, Karaminis et al. (2016) demonstrated atypicalities in prior knowledge in autism more formally, in the context of temporal reproduction, using a Bayesian computational model for central tendency (Cicchini, Arrighi, Cecchetti, & Burr, 2012). The computational model proposed that central tendency reflects the integration of noisy temporal estimates with prior knowledge representations of a mean stimulus. This integration serves to reduce overall error and, crucially, is flexible; the noisier the sensory estimates, the greater the reliance on prior knowledge. Karaminis and colleagues contrasted the performance of autistic and typical children completing a time interval reproduction task (measuring central tendency) and a temporal discrimination task (assessing temporal resolution) to the predictions of the Bayesian model. Computational simulations suggested that central tendency in autistic children was much less than that predicted by computational modeling given the poor temporal resolution of these children. Autistic children presented with a much less flexible use of priors across development compared with typically developing children.

In the current study, we provided another test of the account of Pellicano and Burr (2012) using a well-established perceptual expectation, namely the “light-from-above” prior. The patterns of light and shade on and around an object provide uncertain cues for the brain to determine its shape and position in space. To resolve this ambiguity, the visual system must assume a light source location (Adams, 2007, Gerardin et al., 2007, Mamassian and Landy, 2001). The light-from-above prior weights inference toward assuming that a light source is located above the scene observed, as is most commonly experienced. The presence of a light-from-above prior is demonstrated when an image lit from above is rotated by 180°, so that shading previously perceived as indicating convexity will indicate concavity (and vice versa). Simple rotation changes interpretation (e.g., Mamassian & Goutcher, 2001). A perceptual bias toward light from above is also demonstrated in visual search; observers can identify shapes compatible with light from above significantly faster than the same shapes reversed (i.e., compatible with light from below), whereas shapes suggesting a light source from the side are recognized both more slowly and less accurately (Kleffner & Ramachandran, 1992). A further characteristic of the light-from-above prior is that it is biased slightly to the left of vertical (Gerardin et al., 2007, Mamassian and Goutcher, 2001, Sun and Perona, 1996, Thomas et al., 2010), although there is considerable individual variation in the location of the prior (e.g., Adams, 2007, Champion and Adams, 2007, Mazzilli and Schofield, 2013, Morgenstern et al., 2011). While the preference for an overhead light source is assumed to have an environmental origin, the source of the leftward bias is not well understood (see Mamassian & Goutcher, 2001, for a discussion). The light-from-above prior strongly influences depth perception and assists in reconstructing three dimensions (objects/scenes) from two-dimensional retinal images.

Here, we examined the use of this robust and well-characterized light-from-above prior in autistic children. We also investigated age-related differences in the use of the prior in autistic and typical children. To address these aims, we assessed autistic and typical children of similar age and ability on a shape judgment task designed to reveal individual differences in implicit judgments of light source location. Specifically, we adapted the seven-hexagon stimulus developed by Andrews, Aisenberg, d’Avossa, and Sapir (2012), which was used to demonstrate an effect of cultural differences (reading direction) on location of the prior and, therefore, was thought to be sufficiently sensitive to potential group-level variations. Limited shading information in the stimulus delivered a high level of ambiguity about depth, ensuring that perceptual inference was required to resolve shape and a light source location would need to be assumed.

We sought to investigate whether autistic children would resolve the ambiguous shape-from-shading information in our stimulus using an assumption of light from above. Specifically, we tested whether autistic children would show the bias in stable conditions designed to encourage reliance on prior experience of lighting conditions. An attenuated light-from-above prior, in line with the hypothesis of Pellicano and Burr (2012), might result in more mixed shape judgments according to rotation of the stimulus by autistic children than by typical children, with fewer compatible with light from above. Because priors are thought to smooth over neural noise, attenuated priors in the autistic group might also lead to less stable perception of convexity or concavity, leading to more varied (or less confident) interpretations. Our test purposefully excluded signals that might compete with the light-from-above prior, such as a visible light source (Morgenstern et al., 2011), to determine whether the prior is fundamentally intact and develops similarly in autistic children compared with typical children.

## Method

### Participants

In total, 18 autistic children (16 boys) and 18 typical children (12 boys), all between 7 and 14 years of age, took part in this study. Children were recruited via community contacts. All autistic children had been previously diagnosed with an autism spectrum condition by independent clinicians and scored above the threshold for an autism spectrum disorder on either the Autism Diagnostic Observation Schedule–Second Edition (ADOS-2) or the Lifetime version of the Social Communication Questionnaire (SCQ) (see Table 1 for scores). All typically developing children scored below the cutoff for autism on the SCQ (score of 15; Rutter, Bailey, & Lord, 2003), reflecting the absence of clinically significant autistic features. All children had normal or corrected-to-normal visual acuity, as reported by their parents.

**Table 1:** Participant characteristics.

	Typical children	Autistic children
*n* (boys:girls)	18 (12:6)	18 (16:2)
Age (years)	10.14 (1.76)	10.19 (2.44)
Verbal IQ^a^	105.94 (15.36)	101.11 (15.32)
Performance IQ^a^	99.11 (19.83)	104 (12.95)
Full-scale IQ^a^	102.94 (13.46)	104.72 (14.41)
SCQ^b^	4.59 (3.32)	20.29 (7.15)
ADOS-2^c^		10.65 (3.87)
ADOS-2^c^ CSS^d^		6.23 (1.92)

*Note.* Values are means (and standard deviations) except in the first row.

a Wechsler Abbreviated Scales of Intelligence (WASI-2; Wechsler, 2011).

b Social Communication Questionnaire (SCQ; Rutter et al., 2003).

c Autism Diagnostic Observation Schedule–Second Edition (ADOS-2; Lord et al., 2012).

d ADOS-2 Calibrated Severity Score (CSS; Gotham et al., 2009, Hus and Lord, 2014).

The groups were matched in terms of age, *t*(34) = 0.06, *p* = .95, verbal IQ, *t*(34) = 0.94, *p* = .35, performance IQ, *t*(34) = 0.88, *p* = .39, and full-scale IQ, *t*(34) = 0.38, *p* = .70, as measured by the Wechsler Abbreviated Scales of Intelligence–Second Edition (WASI-2; Wechsler, 2011) (see Table 1 for scores). All children obtained full-scale IQ scores of 70 or above and, thus, were considered to be cognitively able.

An additional 15 typical adults (14 female; 20–35 years of age), recruited from the university, were tested to establish parameters for adult performance in our task. One additional adult was tested but excluded from the analysis because the estimated light source direction (see “Measurements” section below) lay more than 2 standard deviations to the left of the group average. Removing this outlier did not change the results reported here.

### Ethics statement

The study was conducted in accordance with the principles laid out in the Declaration of Helsinki. Ethical approval was granted by the faculty research ethics committee of the university (FPS456). Parents of all children gave their informed written consent prior to the participation of their children in the project, and children gave their verbal assent.

### Stimuli

**Fig. 1:**
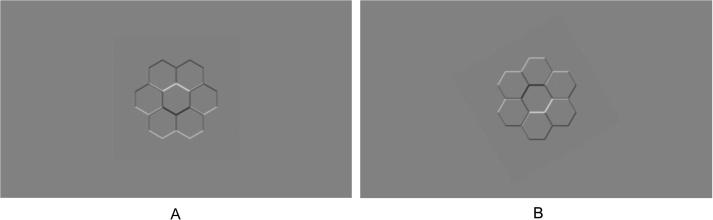
Stimulus with light and shadow as though lit from directly above (A) and rotated + 150° to maximize the appearance of concavity in the central hexagon according to mean adult priors (B).

Following Andrews et al. (2012), the stimulus comprised seven tessellated gray-scale hexagons on a gray-scale background (RGB = 127, 127, 127), with shading on the inner and outer edges of the shapes (see Fig. 1) providing ambiguous cues to depth. Each shape was 2.5° of visual angle, with an intermediate level of blur. The stimulus was rotated by 360° in 15° increments, compatible with different light source locations. We presented 24 rotations in a randomized order in order to estimate the light source direction most consistent with the judgments of convexity and concavity by children. Each rotation was presented five times, yielding a total of 120 trials.

Stimuli were presented on a Dell Precision laptop screen with 1366 × 768 pixel resolution at a refresh rate of 60 Hz and mean luminance of 60 cd/m^2^. All children viewed the stimuli binocularly at a distance of approximately 57 cm from the screen.

### Procedure

**Fig. 2:**
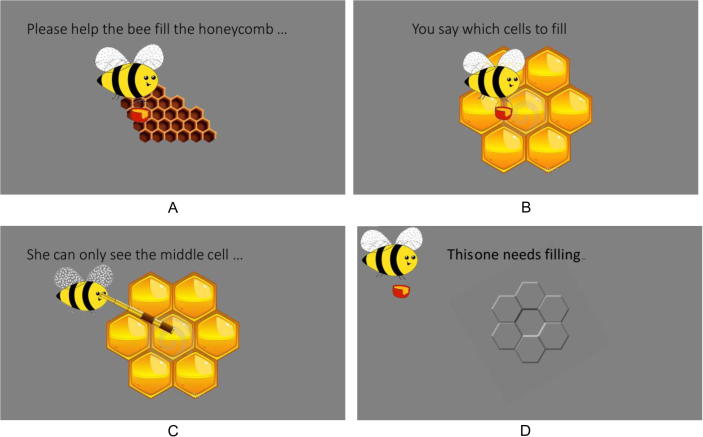
Examples of slides introducing the shape judgment task. Panel D shows the stimulus rotated 150° and is followed by an animation of the bee filling the central cell with honey.

The experiment was written in Matlab using the Psychophysics Toolbox extensions (Brainard, 1997, Kleiner et al., 2007). We measured the implicit judgments of light source location made by children in the context of a child-friendly computer game. During the *introduction phase,* the cover story was introduced showing colored cartoon honeycomb cells and an animated bee. Children were asked to help the bee decide which “cells” needed to be filled with honey (Fig.2A and B). Children were told that they would collect points for their decisions and win a small gift with more than 500 points. Dummy points were also provided to maintain children’s interest. To encourage children to distinguish between cells they perceived as empty and those they saw as full, they were also asked not to “waste” honey when cells were “full.” We also showed the bee with a telescope looking at the central hexagon (Fig.2C) to ensure that answers were given for this cell only.

After the introduction to the story, children were taken through the *demonstration phase.* The cartoon honeycomb was replaced with the hexagon stimulus, which appeared at a rotation of 150° so that the central cell would be most likely to be perceived as concave, according to the known mean prior for adults (Fig.2D). Children were told that this needed filling with honey, which was demonstrated by the bee in an animation. Two demonstration trials followed, using the exact procedure followed in later test trials. The stimulus appeared with the prompt, “Does this one need filling?” Color-coded “yes” and “no” prompts appeared below the question to the left and right of the screen, respectively, in order to match the location of the response keys (letters “a” and “l” on the keyboard). These keys were also labeled “Y” and “N” using sticky labels matched in color to the font of the on-screen response options (yellow for yes and blue for no). Children were asked to press a key to indicate their response, with left (yes) indicating perceived concavity and right (no) indicating perceived convexity. The first demonstration trial again showed the rotation most likely to be perceived as concave, and children were invited to respond. A “yes” response was required to proceed. The second demonstration trial showed the stimulus rotated 330°, the position most likely to be perceived as convex. This required a “no” response and was followed by the caption “No! That one’s full!” Children could repeat the demonstration trials if required, but there were no such further trials in order to avoid influencing subsequent responses (Stone, Kerrigan, & Porrill, 2009). All children produced correct responses for both trials.

The *test phase* commenced immediately following the demonstration phase. An animated bee hovered on the screen (2 s) as a fixation point. Children saw the honeycomb stimulus in one of the 24 rotations, the order of which was randomized. They answered the question “Does this one need filling?” by pressing the “yes” or “no” key according to their interpretation of the stimulus shape. Trials were self-paced, so that each response triggered the next fixation bee. Children completed three blocks of 40 test trials, yielding a total of 120 trials. Dummy scores and general encouragement were provided at the end of each block.

### General procedure

Children were tested individually in a quiet room at the university. The room was lit only by the computer screen, so that no environmental cues were available to influence children’s perceptions of lighting direction (Morgenstern et al., 2011). Testing on the experimental task lasted 10–15 min. The WASI-2 (and the ADOS-2 for autistic children) was administered to children in later sessions.

### Measurements

For each participant, we first calculated the proportion of convex judgments (“no” judgments in our game) for each stimuli direction. Following Andrews et al. (2012), the relation between the proportion of convex judgments (“no” judgments in our game) and stimulus orientation was estimated for each participant using a multivariate logistic regression:

(1) $$p(C|\theta )=1/(1+e^{-f(\theta )}])\text{,}$$

where for a given stimulus direction *θ*, *f*(*θ*) was a series of sine and cosine functions,

(2) $$f(\theta )=\alpha_{0}+\alpha_{1}\cos\theta +\beta_{1}\sin\theta \text{.}$$

We then estimated the light source direction implied by the judgments of each participant as

(3) $$O_{\text{light}\_\text{source}}=\tan^{-1}(\beta_{1}/\alpha_{1})\text{.}$$

## Results

**Fig. 3:**
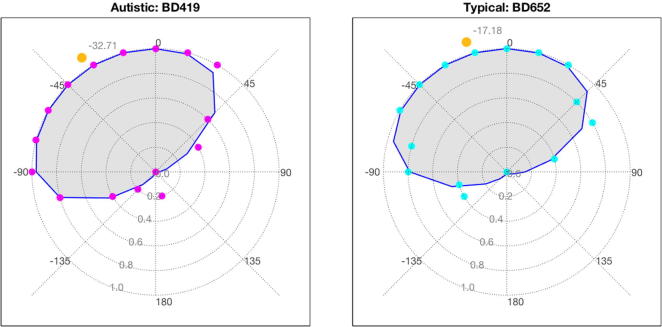
Example data for one autistic participant and one typical child participant. Magenta and cyan dots indicate the proportion of convex judgments for the orientation values tested for one autistic participant (left) and one typical participant (right). Blue lines indicate the predictions of the fitted bimodal distribution. Yellow dots show the estimate of the light source biases for these two participants. (For interpretation of the references to color in this figure legend, the reader is referred to the Web version of this article.)

Fig. 3 shows example results from one autistic and one typical child participant. The plots show two-dimensional psychometric functions on polar axes, plotting percentage convex as a function of rendered orientation. In both cases, the judgment tends to be convex for orientations left of vertical and concave for orientations to the right; this is consistent with a light-from-above interpretation, specifically with a light-from-above left interpretation, in line with previous research (Gerardin et al., 2007, Sun and Perona, 1996, Thomas et al., 2010). The average orientation [estimated from Eq. (3)] is indicated by the orange circle: −32° for the autistic child and −17° for the typical child.

**Fig. 4:**
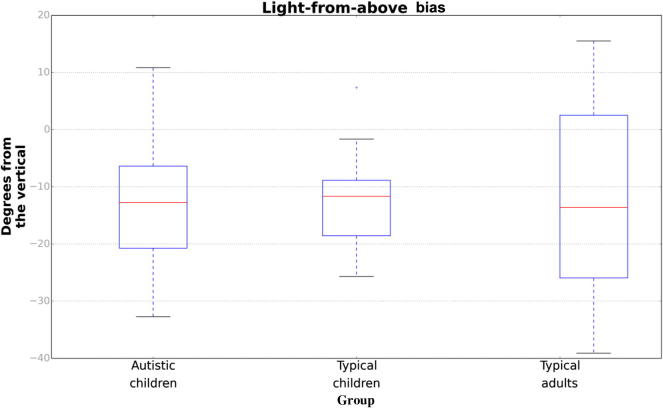
Light source bias estimates for autistic children, typical children, and typical adults estimated from Eq. (3). Negative values correspond to biases to the left of the vertical. Red bands indicate median values; boxes extend from lower to upper quartile values; and whiskers show the full range of values. (For interpretation of the reference to color in this figure legend, the reader is referred to the Web version of this article.)

Fig. 4 shows group averages for the estimated light source direction biases for the three participant groups. As expected (cf. Gerardin et al., 2007, Sun and Perona, 1996, Thomas et al., 2010), average light source biases were to the left of vertical (autistic children: *M* = −12.67°, *SD* = 12.51; typical children: *M* = −12.50°, *SD* = 7.87; adults: *M* = −12.06°, *SD* = 16.72) and were significantly lower than zero for all groups [autistic children: *t*(17) = −4.44, *p* < .001; typical children: *t*(17) = −6.72, *p* < .001; adults: *t*(14) = −2.79, *p* = .01]. A one-way analysis of variance (ANOVA) with group (autistic children, typical children, or adults) as a between-participants factor revealed no significant effect of group on the magnitude of the light bias, *F*(2, 48) = 0.10, *p* = .99. We also examined the data by performing a Bayesian one-way ANOVA using JASP software (Version 0.8.0.0; JASP Team, 2016) and estimating a Bayes factor using Bayesian information criteria (Wagenmakers, 2007). The Bayes factor allowed for a comparison of the fit of our data under the null hypothesis and the alternative hypothesis. The estimated Bayes factor (null/alternative) suggested that our results were 6.56:1 in favor of the null hypothesis, that is, 6.56 times more likely to occur under a model *without* an effect of group on the magnitude of the light-from-above bias rather than a model *with* an effect of group. Our data, therefore, provided substantial evidence (Wetzels et al., 2011) that adults, typical children, and autistic children interpreted the shape of the stimulus in a similar way in that they used a light-from-above prior to a similar degree.

To investigate potential age-related differences in the formation of the prior, we examined the relationship between chronological age and the magnitude of the light bias. There was no significant relationship between age and bias for either group (autistic: *r* = −.24, *p* = .33; typical group: *r* = .03*, p* = .90). There was also no significant relationship with ability, as measured by full-scale IQ scores on the WASI-2, for either group of children (autistic: *r* = −.10*, p* = .95; typical group: *r* = .23*, p* = .36), or with autistic symptomatology, as measured by the ADOS-2 severity scores of children (*r* = −.13, *p* = .61) (Gotham et al., 2009, Hus and Lord, 2014).

We also examined whether the judgments of younger children were influenced by a preference for convexity, as suggested by Thomas et al. (2010), by conducting a Pearson correlation analysis between participants’ percentage of convex judgments (across all responses) and age. However, we found no significant relationship between age and preference to interpret the shape as convex for either group of children (typical: *r* = .05, *p* = .84; autistic: *r* = −.02, *p* = .93).

## Discussion

This study investigated whether autistic and typical children apply a light-from-above prior similarly to interpret shape from shading in conditions where shading cues are ambiguous. The mean light-from-above prior for the children seen in this study was approximately −13° (above and slightly to the left). We found no significant difference in assumed light source location between autistic and typical children. Judgments of depth by autistic children were influenced by a light-from-above prior—that is, they preferentially assumed a light source above and to the left of the stimuli—to a similar extent as those by typical children. Given that all children reported that it was easy to decide whether the cell should be filled, it appears that they used their priors to help resolve noise and ambiguity and achieve stable percepts. This finding is consistent with a recent report suggesting that priors for eye gaze direction—where gaze is more likely to be perceived as direct in conditions of uncertainty—are intact in autistic adults (Pell et al., 2016).

In our task, environmental conditions other than rotation were held constant, so that inference relied on participants’ existing priors, summing their long-term experience of lighting conditions. We used a task that had previously shown subtle differences between groups whose long-term (cultural) experience of reading direction differed (Andrews et al., 2012), suggesting that it should have been possible to detect differences based on long-term experience between our groups if they were in fact present.

Similar mean levels might nevertheless conceal different group levels of adaptation in the short term to prevailing environmental conditions. Experimental evidence has demonstrated that changes in prevailing environmental conditions can modify light priors in the short term. In the case of typical adults, the light-from-above prior adapts rapidly (Adams et al., 2004, Adams et al., 2010, Champion and Adams, 2007), so that training with haptic feedback can change an individual light prior location by approximately 10° with 1.5 h of training (Adams et al., 2004). Participants’ visual judgments on a separate task after training were recalibrated in line with the new prior (Adams et al., 2010, Champion and Adams, 2007), indicating that the prior had been temporarily updated. Similarly, learning an association between a context and its lighting conditions led to contextually appropriate calibration of the light prior (Kerrigan & Adams, 2013), in line with Bayesian updating of the prior probability distribution by context. Flexible adaptation to prevailing conditions was not tested in the current study but is a worthy avenue for future research and a more direct test of the account of Pellicano and Burr (2012).

It is also important to consider precisely how prior knowledge acts on perceptual inference. Although evidence strongly suggests that an assumed light source position is represented early in the visual system and acts on early inference (Champion and Adams, 2007, Lee et al., 2002, Mamassian et al., 2003), perceptual computation also appears to be an interactive process, so that activity in the early visual cortex may nevertheless take into account behavioral experience and higher order perceptual saliency (Lee, 2003, Lee et al., 2002). Champion and Adams (2007) found evidence of both early and late influence of the light-from-above prior on perceptual inference. Although visual haptic training modified the light-from-above prior used in a judgment of shape task, the prior assessed in a visual search task was not affected by the same training (Champion & Adams, 2007). The discrepant effects of the training environment were interpreted as evidence that although training did not touch the “quick and dirty” process of early inference, it influenced later additional stages of visual processing, where recent experience with the world is taken into account. Because our task did not require accounting for recent experience, the mean priors we found may reflect the influence of the prior on the initial stage of visual processing alone, leaving open the possibility of reduced influence of priors during later stages of visual processing for autistic children. Future research should test how far the priors of children adapt to prevailing environmental conditions.

Our findings also show that the two groups of children performed similarly to adults. Furthermore, we found no age-related changes in the use of the light-from-above prior, at least in children between 7 and 14 years of age, suggesting that the prior develops early—before 7 years. This would be consistent with recent findings showing that typical children use prior knowledge in magnitude estimations from an early age, in particular for temporal (Karaminis et al., 2016) and spatial (Sciutti, Burr, Saracco, Sandini, & Gori, 2014) interval reproduction. If learning an internal model of the sensory environment is key to the statistical inference process involved in Bayesian perception, and also is critical to learning beyond perception (Fiser, Berkes, Orbán, & Lengyel, 2010), early acquisition of priors, especially robust ones like these, would make sense developmentally. However, Karaminis et al. (2016) found differences between autistic and typical development in time interval reproduction. Autistic children showed significantly less precision in their estimations than matched typical children, with computational simulations suggesting less use of prior knowledge than expected to compensate for this level of imprecision. This result implies differences in the flexible deployment of priors to improve the precision/reliability of estimates. Our task showed no differences between typical and autistic children in the use or development of the light-from-above prior, at least in controlled conditions where competing information is limited (cf. Morgenstern et al., 2011), but the findings of Karaminis and colleagues are another reason to study the flexibility of the light-from-above prior (cf. Adams et al., 2010) in autistic children.

Our findings are in contrast to other research reporting developmental increases in responses consistent with light-from-above prior (Stone, 2011, Stone and Pascalis, 2010, Thomas et al., 2010), although the evidence is not clear-cut. Thomas et al. (2010) found an overall effect of age on children’s interpretation of ambiguous stimuli, so that trials answered assuming light from above increased across childhood. Yet this result was complicated by the measurement of two distinct priors—one for convexity and one for light from above. When the two priors biased interpretation in the same direction, there was little change in use of the light-from-above prior over development; when the priors conflicted, younger children (4- and 5-year-olds) preferred a convex interpretation, whereas older children and adults preferred to assume light from above *if* light could be interpreted as from above and to the left rather than as above right. However, the number of younger children was small (*n* = 7), with some performing at around chance levels. Stone and Pascalis (2010) showed children aged 4–10 years geometric shapes and photographic stimuli, either upright or rotated 180°, reporting increasing levels of interpretation consistent with light from above by age group. Yet the results for symbolic images—where ambiguity is higher and, therefore, the potential influence of the prior is stronger—did not clearly support this interpretation. In particular, the regression analysis for concave stimuli (if lit from above) against age was nonsignificant, whereas that for convex symbols (if lit from above) indicated greater use of the prior for children aged around 7 or 8 years. Overall, these results point to the difficulty of disentangling the influence of light from above and convexity priors and the difficulty of interpreting which factors influenced the performance of younger children.

In sum, we have established that the light-from-above prior is similar in strength in a shape judgment task in school-age autistic and typical children of similar age and ability. Furthermore, contrary to previous reports (Stone and Pascalis, 2010, Thomas et al., 2010), our results suggest a stable level of use of the prior across this age range. Although our methods were developmentally sensitive, with performance not being confounded with linguistic demands (concave and convex), our sample sizes were relatively small and we may have had insufficient power to detect age-related changes in the use or strength of the prior; this is especially important given that priors vary widely between individuals (e.g., Adams, 2007, Champion and Adams, 2007, Mazzilli and Schofield, 2013, Morgenstern et al., 2011). One outstanding question is whether the light-from-above prior is just as adaptable as it is in adults for typically developing children—but especially for children on the autism spectrum.
